# Acidophilic algae isolated from mine-impacted environments and their roles in sustaining heterotrophic acidophiles

**DOI:** 10.3389/fmicb.2012.00325

**Published:** 2012-09-11

**Authors:** Ivan Ňancucheo, D. Barrie Johnson

**Affiliations:** ^1^School of Biological Sciences, Bangor UniversityBangor, UK; ^2^Agriculture of Desert and Biotechnology, Universidad Arturo PratIquique, Chile

**Keywords:** acidophilic algae, *Chlorella*, *Euglena*, glycolic acid, microbial interactions, mine waters, monosaccharides

## Abstract

Two acidophilic algae, identified as strains of *Chlorella protothecoides* var. *acidicola* and *Euglena mutabilis*, were isolated in pure culture from abandoned copper mines in Spain and Wales and grown in pH- and temperature-controlled bioreactors. The *Chlorella* isolate grew optimally at pH 2.5 and 30°C, with a corresponding culture doubling time of 9 h. The isolates displayed similar tolerance (10–50 mM) to four transition metals tested. Growth of the algae in liquid media was paralleled with increasing concentrations of dissolved organic carbon (DOC). Glycolic acid was identified as a significant component (12–14%) of total DOC. Protracted incubation resulted in concentrations of glycolic acid declining in both cases, and glycolic acid added to a culture of *Chlorella* incubated in the dark was taken up by the alga (~100% within 3 days). Two monosaccharides were identified in cell-free liquors of each algal isolate: fructose and glucose (*Chlorella*), and mannitol and glucose (*Euglena*). These were rapidly metabolized by acidophilic heterotrophic bacteria (*Acidiphilium* and *Acidobacterium* spp.) though only fructose was utilized by the more fastidious heterotroph “*Acidocella aromatica.*” The significance of algae in promoting the growth of iron- (and sulfate-) reducing heterotrophic acidophiles that are important in remediating mine-impacted waters (MIWs) is discussed.

## Introduction

Mining of metals and coal can impair the environment in many ways. One of the most widely documented is the generation of mine-impacted water bodies (MIWs; drainage streams and pit lakes) that are characteristically acidic (sometimes extremely so) and which contain elevated concentrations of iron and other transition metals, aluminium, sulfate, and sometime arsenic. These constitute an “extreme” environment, which is hostile to most life forms (reviewed in Johnson, 2009). In the most severe cases, indigenous organisms are exclusively microbial and predominantly prokaryotic. Eukaryotic microorganisms, including acidophilic and acid-tolerant species of microalgae, fungi and yeasts, protozoa and rotifera, have, however, been reported in MIWs on a number of occasions (e.g., Baker et al., 2004; Aguilera et al., 2007; Das et al., 2009).

Primary production in MIWs is mediated by autotrophic microorganisms that use either solar or chemical energy to fuel carbon dioxide fixation. In subterranean locations, chemolithotrophic acidophilic bacteria and archaea that use ferrous iron, reduced sulfur (and possibly hydrogen) as electron donors are the sole agents of primary production (e.g., Bond et al., 2000; Johnson, 2012). Mechanisms of carbon fixation and other physiological characteristics of bacteria such as *Acidithiobacillus* spp. and *Leptospirillum* spp. have been well studied, not only because these mineral-oxidizing acidophiles are generally acknowledged to be the most important micro-organisms involved in the genesis of MIWs, but also because the same bacteria are considered to be the most significant agents of metal extraction in commercial “biomining” operations (Rawlings and Johnson, 2007). However, where solar energy is available, net carbon fixation by acidophilic microalgae may exceed that of chemolithotrophic bacteria, since the most abundant chemical energy source in the most acidic mine waters (ferrous iron) is a relatively poor electron donor in terms of free energy (ΔF_298_ of −73 kJ; Kelly, 1978).

The biodiversity of validated species of acidophilic and acid-tolerant algae is relatively limited (reviewed in Novis and Harding, 2007). Micro-algae reported to be metabolically active in metal-rich, highly acidic environments include some Chlorophyta, such as *Chlamydomonas acidophila* and *Dunaliella acidophila*, Chrysophyta, such as *Ochromonas* sp., and Euglenophyta, such as *Euglena mutabilis*. Some diatoms, including several *Eunotia* spp., have also been found to colonize extremely acidic waters. Filamentous algae, identified as *Zygnema circumcarinatum* and *Klebsormidium acidophilum*, have been found in extremely acidic (pH <3) mine waters in Spain and New Zealand, respectively (Novis and Harding, 2007; Rowe et al., 2007). Moderately thermophilic and acidophilic Rhodophyta (*Cyanidium caldarium, Galdieria sulfuraria*, and *Galdieria maxima*) are frequently encountered in acidic waters in geothermal areas (Toplin et al., 2008). In contrast to acidophilic autotrophic bacteria, there have been few reports of laboratory studies of axenic cultures of acidophilic microalgae, presumably due to difficulties in obtaining cultures of these eukaryotes that are free of bacteria. Axenic cultures are necessary, for example, to identify and quantify the amounts of organic carbon released by acidophilic algae, a phenomenon that has previously been reported for chemolithotrophic acidophiles (e.g., Ňancucheo and Johnson, 2010).

Macroscopic growths of acidophilic algae have been reported in various streams and rivers in the Iberian Pyrite Belt (IBP) in south-western Spain. Aguilera et al. (2007) found that the diversity of indigenous algae in biofilm growths was related to the acidity and metal contents of sites within the Rio Tinto, and that algal populations displayed seasonal trends. Initially, biofilm growths were dominated by flagellated green algae (*Dunaliella* or *Chlamydomonas*), and *Euglena*. Later sessile species of algae such as *Chlorella* and *Cyanidium* appeared, followed lastly by filamentous algae (*Zygnemopsis* and *Klebsormidium*). Elsewhere in the IBP, Rowe et al. (2007) reported that an open drainage channel at an abandoned copper mine (Cantareras) contained a thick (~12 cm) microbial mat, the upper layer (~2.5 mm) of which was green colored and contained both unicellular (*Euglena* and *Chlamydomonas*) and filamentous (*Zygnema*) micro-algae. The mat under the algal layer was bacterial, and dominated by heterotrophic acidophiles (including species of ferric iron- and sulfate-reducing bacteria). With increasing distance from the mine adit, algae were rare or absent, and this correlated with a much less thick (or absent) microbial mat within the drain channel. The inference was made that the autotrophic algae were providing the organic materials that sustained the heterotrophic acidophiles in the microbial mat, as the concentration of dissolved organic carbon (DOC) in the mine water at its point of discharge was very low (~1 mg/L).

Here we describe the isolation, in pure culture, of strains of two acidophilic algae (*Chlorella protothecoides* var. *acidicola* and *Euglena mutabilis*) from MIWs in Spain and Wales, the nature of their organic exudates and lysates, and the significance of the latter in sustaining heterotrophic bacteria in acidic mine waters.

## Materials and methods

### Origins, isolation, and cultivation of acidophilic algae

**Figure 1:**
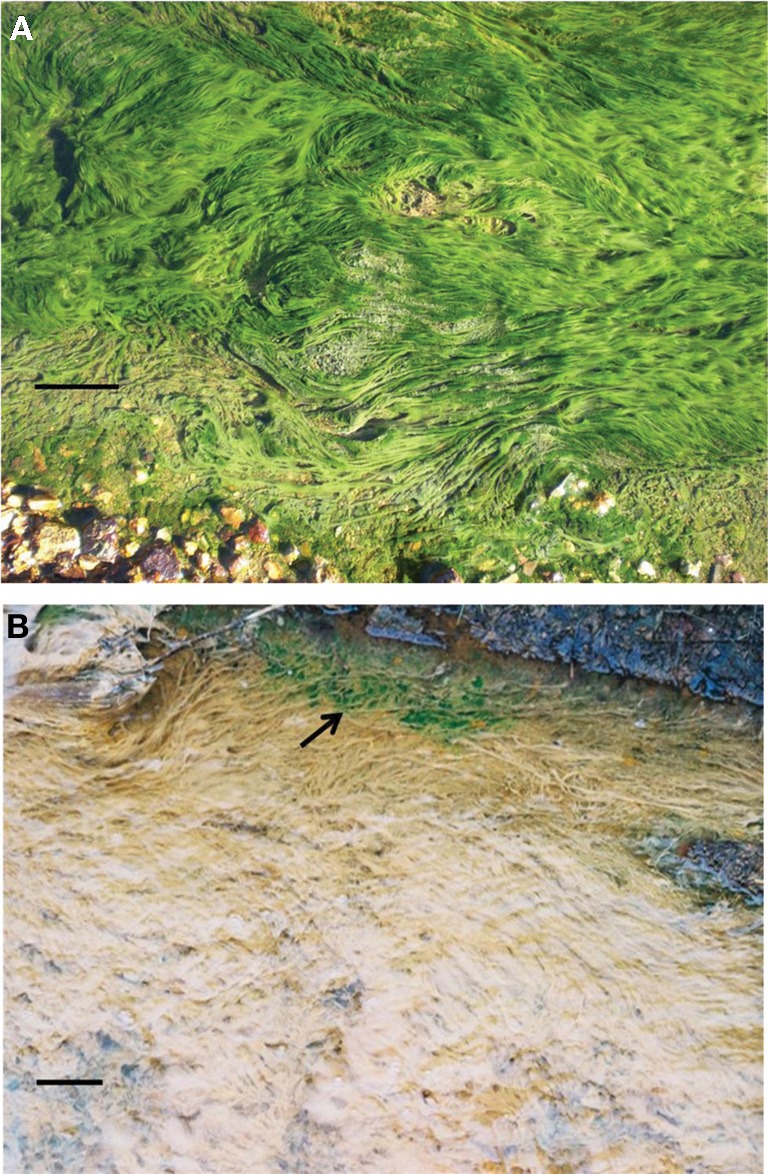
**Surface algal growths on a microbial mat in a stream draining the abandoned Cantareras copper mine, Spain (A); acid streamers colonized with *Euglena mutabilis* (arrowed) in a stream draining the abandoned Mynydd Parys mine, Wales (B).** The solid bar represents ~10 cm in both images.

The two acidophilic algae investigated in the present study were isolated from extremely acidic (pH 2.5–2.6) MIWs draining abandoned copper mines in Europe. *Chlorella protothecoides* var. *acidicola* was isolated from surface growths of a microbial mat at Cantareras, Spain (Rowe et al., 2007; Figure 1A) while *Euglena mutabilis* was isolated from green streamer-like growths at Mynydd Parys, Wales (Coupland and Johnson, 2004; Figure 1B). In both cases, small algal growths were removed using sterile tweezers, suspended in acidic (pH 2.5) basal salts and dispersed by vortexing. Cell suspensions were then streaked onto a solid medium containing acidified (pH 2.5) basal salts and trace elements (Wakeman et al., 2008) supplemented with 100 μM ferrous sulfate (aBS/TE), and gelled with 0.5% (w/v) agarose (Sigma type I). The pH of the gelled medium, measured with a flat bottomed combination pH electrode (Russell pH, UK) was ~2.8. Plates were incubated at 22°C under a light bank with constant illumination (70 μmol of photons m^−2^ s^−1^). After ~20 days, small green-colored colonies were apparent. These were re-streaked onto fresh plates, and single colonies from these placed into aBS/TE liquid medium (25 mL in 100 mL conical flasks) and incubated under light. Microscopic examination showed that bacteria, as well as single eukaryotic cell morphologies, were present, in grown cultures. To eliminate the bacteria, the micro-algae were cultivated in aBS/TE medium containing various mixtures of antibiotics [ampicillin (100, 300, and 500 μg/mL) supplemented (or not) with 100 μg/mL of streptomycin]. This procedure was repeated until no bacteria were observed by phase contrast microscopy and when algal cultures were inoculated into media that support the growth of acidophilic bacteria (Johnson and Hallberg, 2007). Aliquots of purified liquid cultures were streak-inoculated onto solid medium, and single colonies from these placed into liquid aBS/TE medium containing no antibiotics. Cultures were checked routinely for the presence of bacteria throughout the course of the experiments.

The identities of the algal isolates were confirmed by amplification and sequencing of their 18S rRNA genes. Genes were amplified by PCR (30 cycles; denaturation for 30 s at 95°C, annealing for 30 s at 55°C, and elongation at 72°C for 90 s, followed by 10 min period at 72°C). The primers used, EukF (ACCTGGTTGATCCTGCAG) and EukR (TGATCCTTCYGCAGGTTCAC), were modified versions of those described by Medlin et al. (1988). Gene sequences were determined using a Beckman Coulter dye terminator cycle sequencing kit and a CEQ8000 Genetic Analysis System (Beckman Coulter, UK) and were compared with those in public databases using BLAST.

### Bioreactor cultures of acidophilic algae

Purified cultures of the two acidophilic algal isolates were grown in aBS/TE liquid medium in a 2 L (working volume) FerMac 200 modular bioreactor (Electrolab, UK) under constant illumination. For routine cultivation, the *Chlorella* isolate was grown at pH 2.5 and at 25°C, and cultures were aerated at 200 mL/min and stirred at 75 rpm. The *Euglena* isolate was grown under the same conditions, but without stirring. To determine the effects of pH and temperature on the growth of the *Chlorella* isolate, cultures were grown at: (1) a fixed temperature of 25°C and varying (and fixed) pH (2.0–3.5), and (2) a fixed pH of 2.5 and temperature ranging from 22 to 35°C. Samples were removed from the reactor at regular intervals and cell numbers determined initially both by enumerating cells (using a Helber counting chamber marked with Thoma ruling (Hawksley, UK) and viewed with a Leitz Labolux phase contrast microscope, at a magnification of ×400) and by measuring the optical densities (OD) of cultures at 600 nm. Since the two measurements were found to be highly correlated (data not shown), only OD measurements were made in later experiments. Culture doubling times were evaluated from semi-logarithmic plots of cell number increase against time. Attempts to carry out parallel growth response experiments with the *Euglena* isolate were not successful as mechanical agitation (stirring) of the bioreactor was found to impede the growth of this alga (possibly due to physical damage to the cells), and consequently this isolate did not grow as dispersed planktonic phase cells, precluding accurate determination of cell numbers.

### Effect of some transition metals on the growth of algal isolates

Isolates were grown in aBS/TE liquid medium (5 mL aliquots in 25 ml universal bottles) containing different concentrations (0, 10, 50, 100, and 200 mM) of transition metals (Cu^2+^, Fe^2+^, Fe^3+^, Ni^2+^, or Zn^2+^, all added as sulfates). The cultures were set at pH 2.5 and were incubated at 22°C, shaken (100 rpm) for 30 days. Growth of the micro-algae was assessed by microscopic examination of the cultures.

### Dissolved organic carbon in cultures of algal isolates

Isolates were grown in a bioreactor for 60 days (the *Chlorella* isolate) or 90 days (the *Euglena* isolate), the cultures removed and cell-free liquors obtained by centrifugation (10,000× g; 15 min) followed by filtration through 0.2 μm (pore size) cellulose nitrate membrane filters (Whatman, UK). Concentrations of total DOC in cell-free culture liquors were measured using a LABTOC DOC analyzer (Pollution and Process Monitoring, UK). Glycolic acid and other aliphatic acids were determined using a combination of ion chromatography and colorimetry (Ňancucheo and Johnson, 2010). Carbohydrates and amino acids were determined using a Dionex ICS 3000 ion chromatograph fitted with an ED amperometric detector. Separation of sugars was carried out on a Dionex CarboPac MA1 column with a CarboPac MA1 guard column, eluted with 0.25 mM sodium hydroxide (0.4 mL/min), and amino acids were separated on a Dionex AminoPAC PA10 column.

### Metabolism of glycolic acid by the *Chlorella* and *Euglena* isolates

The *Chlorella* isolate was grown in a bioreactor, as described above, for 20 days. On day 20, the light source was removed, and on day 22 glycolic acid was added to give a concentration of 0.5 mM in the reactor vessel. The light source was reinstated on day 25. Samples were withdrawn at regular intervals to measure the concentrations of total DOC and glycolic acid, and to determine OD.

### Metabolism of algal DOC by acidophilic heterotrophic bacteria

The acidophilic, iron-reducing heterotrophic bacteria *Acidiphilium* SJH (Bridge and Johnson, 2000), “*Acidocella* (*Ac.*) *aromatica*” strain PFBC (Coupland and Johnson, 2008) and the type strain of *Acidobacterium* (*Ab.*) *capsulatum* (Kishimoto et al., 1991) were sourced from the *Acidophile Culture Collection* maintained at Bangor University, and assessed for their abilities to metabolize DOC present in the algal cultures. Cell-free culture liquors were prepared as above, adjusted to pH 3.0 with 1 M NaOH, and 20 mL aliquots dispensed into 100 mL conical flasks. These were inoculated (duplicate cultures) with each of the three heterotrophic bacteria, and a fourth set used as sterile controls. Cultures were incubated with shaking (150 rpm) at 30°C for up 6 days and samples were withdrawn at days 0, 3, and 6 to enumerate bacterial cells (total counts, as above) and concentrations of total DOC and monosaccharides.

## Results

### Identification of acidophilic algal isolates

Green-pigmented colonies were observed after 10–14 days on gelled “inorganic” solid media inoculated with surface streamer growths from both Cantareras and Mynydd Parys, and incubated in the light. After a single transfer on solid medium and inoculation into liquid media, cultures of the micro-algae were found to be contaminated with bacteria. These were eliminated by subculturing in the presence of both ampicillin (500 μg/mL) and streptomycin (100 μg/mL). Subsequently, axenic cultures of two algal isolates were maintained in liquid medium. One of these grew as single or small groups of round to oval-shaped cells, ~3–5 μm diameter, while the other occurred as aggregating worm-like cells, ~30–50 μm long, that displayed gliding motility. The identities of these isolates were confirmed by analysis of their 18S rRNA genes (1678 nt and 1778 nt gene length products for the *Chlorella*- and *Euglena*-like isolates, respectively). The smaller algae shared 99% gene similarity with *Chlorella protothecoides* var. *acidicola* (strain 124, the closest related strain in GenBank) which was isolated from acidic (pH 2) water at Pisciarelli, Italy (AJ439399; Huss et al., 2002), while the other shared 99% gene similarity with a strain of *Euglena mutabilis* (ELC 1) isolated from Lake Caviahue, an acidic water body in Argentina (EU090196; Brankatschk et al., unpublished). The 18S rRNA gene sequences of the current isolates have been deposited in GenBank, and have the accession numbers JF694006 (the *Chlorella* isolate) and JF694007 (the *Euglena* isolate).

### Effect of pH and temperature on the growth of *Chlorella* isolate

**Figure 2:**
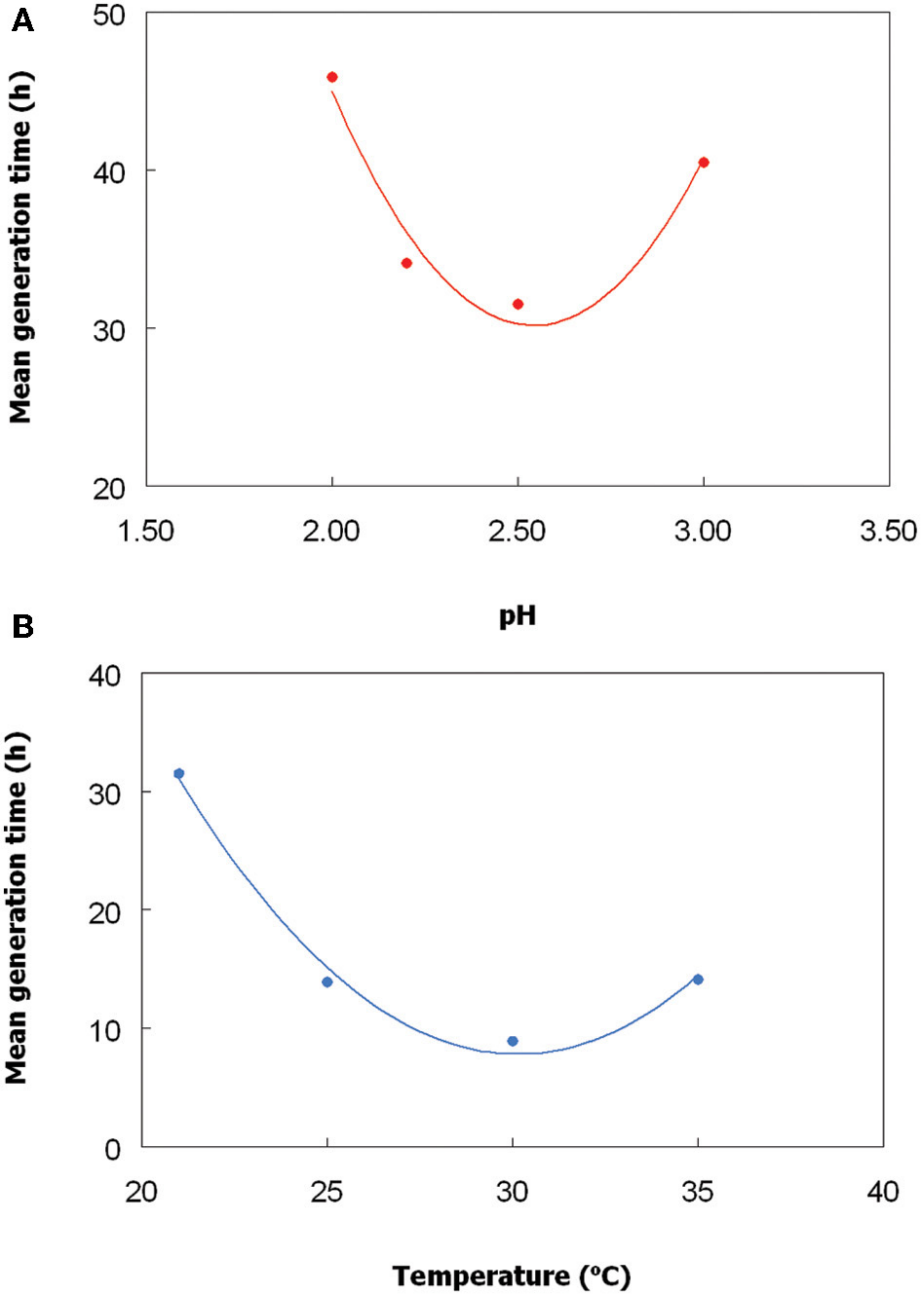
**Effect of (A) pH, and (B) temperature, on growth of the *Chlorella* isolate.** To determine the pH optimum, cultures were grown at 25°C, and to determined temperature optimum, cultures were grown at a constant pH (2.5). The lines shown are best fits based on polynomial analysis of the data points.

The *Chlorella* isolate grew between pH 2 and 3.5, and optimally at ~pH 2.5 (Figure 2A, showing single data points for each pH and temperature tested). No growth was obtained at pH 1.8, and growth above pH 3.5 was not tested. The optimum temperature for growth of this alga was ~30°C (Figure 2B), and growth was strongly inhibited at 40°C. Under optimum conditions of pH and temperature, the *Chlorella* isolate had a culture doubling time of 9 h. The temperature and pH characteristics of the *Euglena* isolate was not determined due to extensive biofilm formation in the growth vessel, though von Dach (1943) had earlier reported growth of a strain of *E. mutabilis* between pH 2.1 and 7.7, and optimum growth between pH 3.4 and 5.4.

### Effects of transition metals on growth

The *Chlorella* and *Euglena* isolates showed similar tolerances to the transition metals tested. Both isolates grew in the presence of 10 and 50 mM, but not 100 mM, of ferrous or ferric iron. Both isolates were more sensitive to copper, zinc, and nickel, with growth being observed in media containing 10 mM but not in 50 mM (or higher concentrations) of these transition metals.

### Organic carbon in batch cultures of *Chlorella*

**Figure 3:**
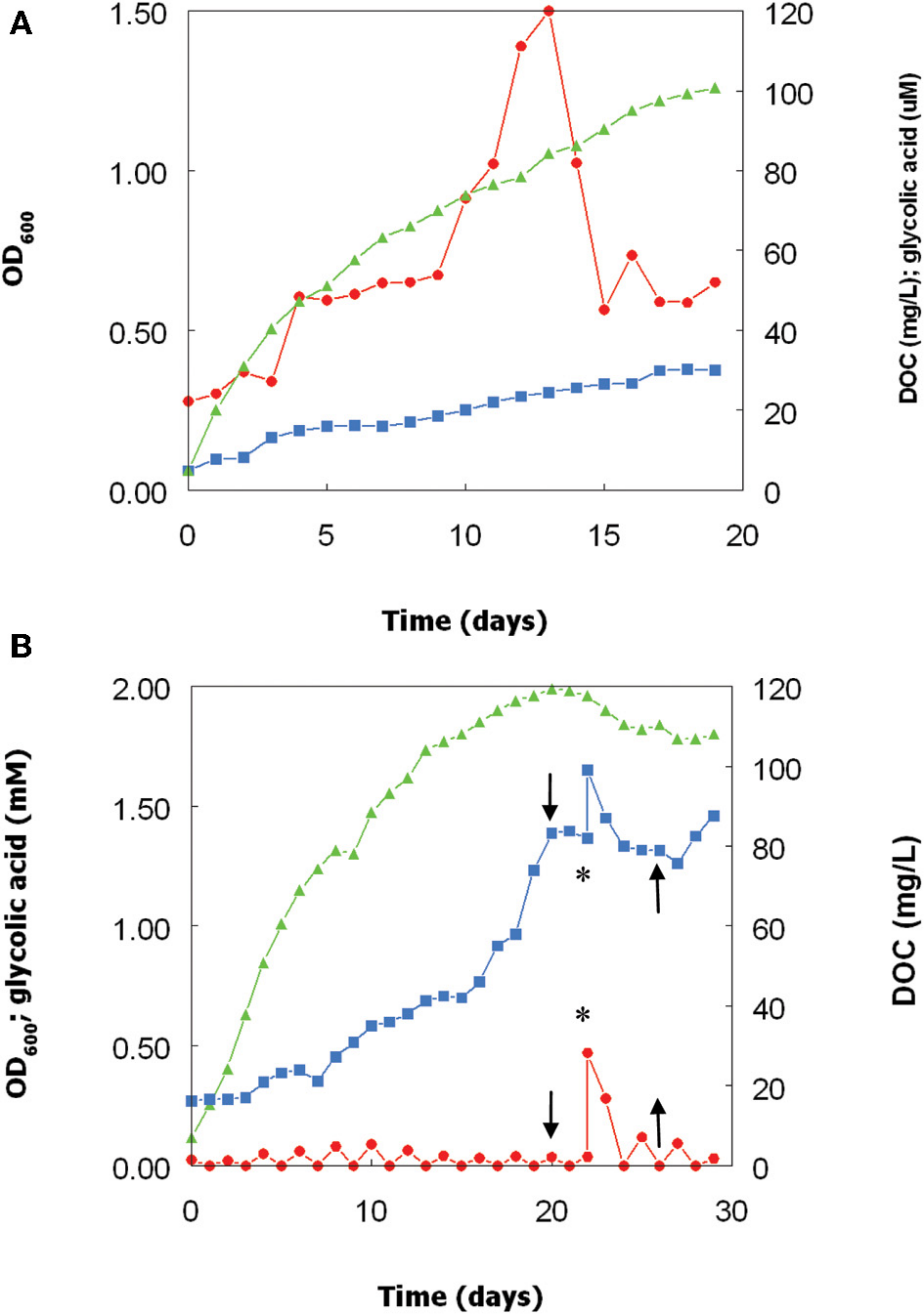
**Changes in optical densities and concentrations of DOC in glycolic acid in cultures of the *Chlorella* isolate, grown at pH 2.5 and 30°C over (A) 19 days, and (B) 29 days.** In the second experiment, the light source was cut off at day 20 and reinstated at day 25 (indicated by the downward and upward pointing arrows, respectively) and glycolic acid added (to 0.5 mM) at day 22 (indicated by the asterisk). Key: (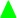), culture optical densities at 600 nm; (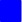) total DOC; (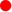) glycolic acid.

Growth of the *Chlorella* isolate at pH 2.5 and 30°C was paralleled by increasing concentrations of DOC, from 4 mg/L at day 0–30 mg/L at day 19 (Figure 3A). During the first 13 days of culture growth, concentrations of glycolic acid also increased, reaching a maximum value of 120 μM (corresponding to ~12% of total DOC in the cell-free culture liquor). From day 13–15, glycolic acid concentrations fell by ~60%, though this was not reflected by a lower DOC values. From day 15, the glycolic acid concentrations remained fairly stable, at a level corresponding to about 4% of total DOC.

A repeat bioreactor experiment was carried out for a more protracted (29 day) period (Figure 3B). *Chlorella* increased more rapidly than over the first 15 days than in the first experiment, and the DOC concentration was ~60% greater at day 15 than in the previous experiment. After day 15, growth of the alga slowed down, but the rate of increase of DOC during this period was greater than previously observed. Removing the light source at day 20 resulted in the immediate cessation of both algal growth (followed by a decline in culture optical density) and accumulation of DOC. Glycolic acid, added to the bioreactor culture at day 22, was found to be quickly removed from solution, with concentrations of both glycolic acid and DOC returning to levels similar to those immediately prior to addition of extraneous glycolic acid by day 25 (Figure 3B). At day 25, the light source was reinstated, which resulted in DOC concentrations again increasing and also, to more limited extent, *Chlorella* biomass (culture OD).

### Organic carbon in batch cultures of the *Euglena* isolate

**Figure 4:**
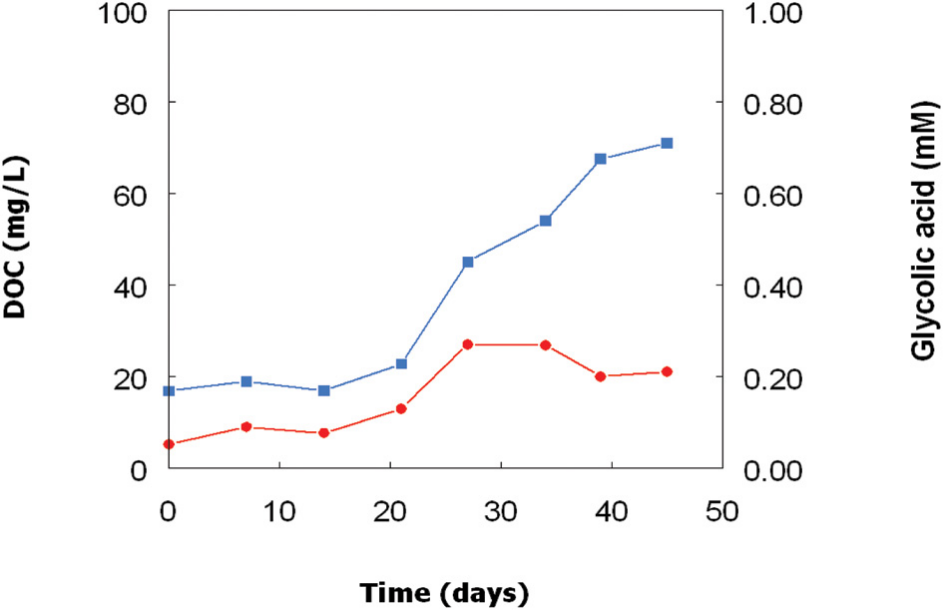
**Changes in concentrations of DOC (**
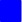
**) and glycolic acid (**
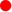
**) in a culture of the *Euglena mutabilis* isolate grown in a bioreactor at pH 2.5 and 30°C**.

As with the *Chlorella* isolate, concentrations of DOC and glycolic acid also increased in bioreactor cultures of the *Euglena* isolate (Figure 4) though, due to extensive biofilm formation in the growth vessel, these changes could not be directly correlated with growth of this acidophilic alga. As with *Chlorella*, glycolic acid concentrations increased initially and then stabilized before declining a little, though not to the same extent as in the *Chlorella* cultures. At day 27, the concentration of glycolic acid in the cell-free culture liquor was equivalent to 14% of the DOC, but this figure fell to ~7% by day 45.

### Identification of other organic compounds in DOC of acidophilic algal cultures

Besides glycolic acid, the only organic compounds identified in cultures of both the *Chlorella* and *Euglena* isolates were monosaccharides. Extensive analysis failed to detect amino acids and other aliphatic acids. Two monosaccharides were detected in each of the two algal cultures—fructose and smaller concentrations of glucose in cultures of *Chlorella*, and mannitol and glucose in cultures of *Euglena*. In both cases, these accounted for smaller amounts of total DOC than the glycolic acid component (Table 1).

**Table 1:** **Monosaccharides identified in cell-free culture liquors of the acidophilic *Chlorella* and *Euglena* isolates, determined after 60 and 90 days of culture incubation, respectively**.

	**Concentration (μM)**	**Concentration (C equivalent; μM)**	**Total DOC (mg/L)**	**Contribution to DOC (%)**
***CHLORELLA* ISOLATE**				
Glucose	5	0.36	167	0.22
Fructose	38.5	2.8	167	1.7
***EUGLENA* ISOLATE**				
Glucose	65	4.7	192	2.5
Mannitol	43	3.1	192	1.6

### Growth of acidophilic heterotrophic bacteria in cell-free culture liquors of *Chlorella* and *Euglena*

**Figure 5:**
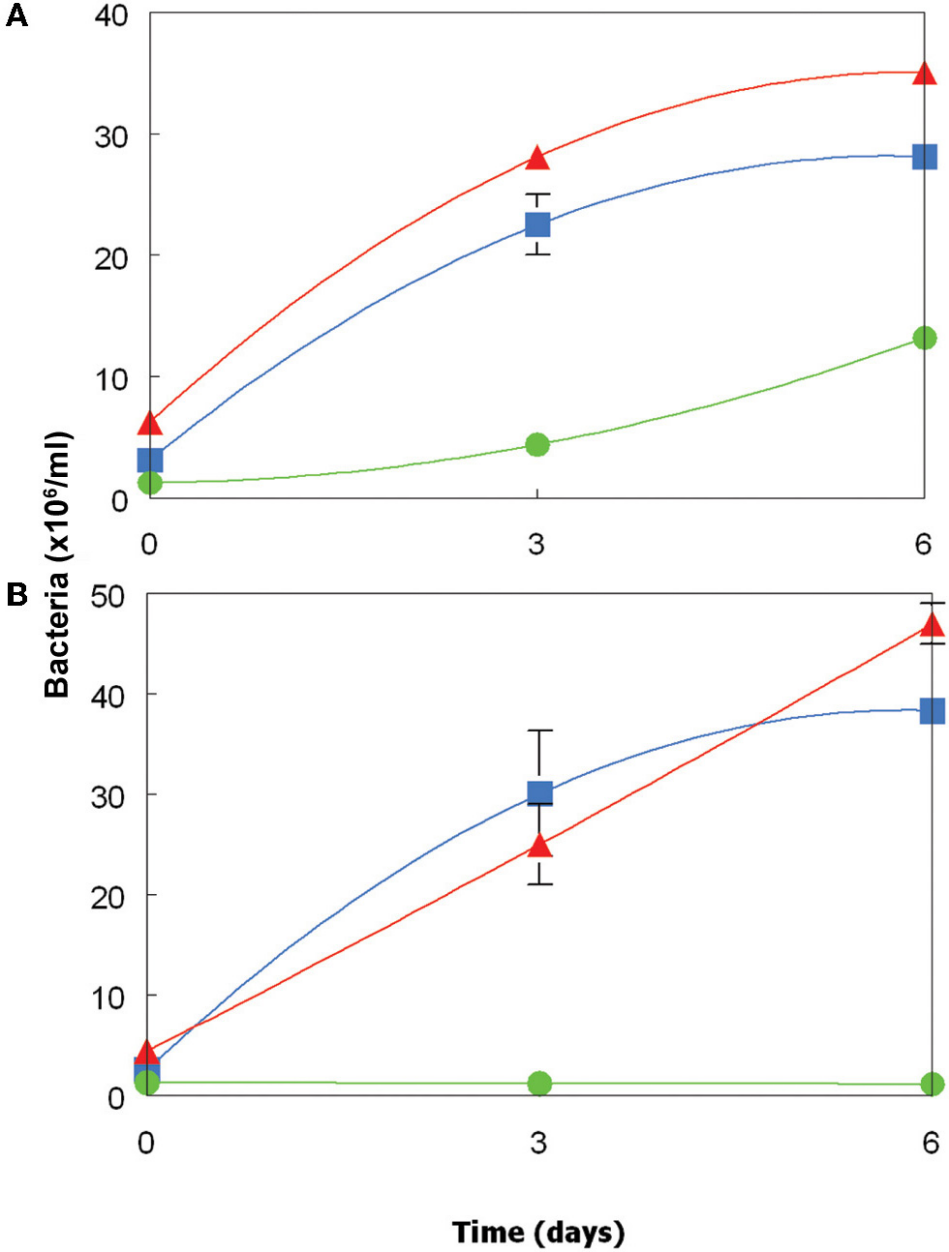
**Growth of acidophilic heterotrophic bacteria in cell-free culture media of (A) the *Chlorella* isolate, and (B) the *Euglena* isolate.** Key: (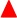) *Acidiphilium* sp. SJH; (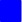) *Acidobacterium capsulatum*^*T*^; (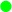) “*Acidocella aromatica*” strain PFBC. Error bars show standard deviations (where not visible, these were smaller than the symbols used for each data point).

Dissolved organic materials originating from both the *Chlorella* and *Euglena* isolates were able to support the growth of representative species of two genera of acidophilic heterotrophic bacteria frequently found in MIWs, *Acidiphilium* and *Acidobacterium*, though the species of *Acidocella* used (“*Ac. aromatica*” strain PFBC) was only found to grow in cell-free culture liquor of *Chlorella* (Figure 5). Growth of heterotrophic acidophiles was accompanied by corresponding decreases in concentrations of DOC and monosaccharides (Table 2). In the case of “*Ac. aromatica*” PFBC, fructose declined to less than detectable concentrations, though those of other sugars were similar after 6 days incubation to initial values. Growth of the different species of heterotrophic acidophiles appeared to correlate with net changes in DOC, with *Acidiphilium* SJH showing the largest increase in cell numbers and greatest change in DOC concentrations during incubation, and “*Ac. aromatica*” PFBC the opposite trend. DOC concentrations in cell-free culture liquors of *Chlorella* declined by between 5 mg/L (for “*Ac. aromatica*” PFBC) and 12 mg/L (for *Acidiphilium* SJH), which was greater than the combined carbon equivalents (3.2 mg/L) of the two monosaccarides (glucose and fructose) that were analysed. Corresponding data for the *Euglena* cell-free culture liquors were 17 mg/L for *Ab. capsulatum* and 22 mg/L for *Acidiphilium* SJH, compared with a combined carbon equivalent of 7.8 mg/L for glucose and mannitol (both of which were >95% metabolized). These data indicate that other components of the DOC were also utilized by the heterotrophic acidophiles, though this was not the case with “*Ac. aromatica*” PFBC cultivated in cell-free culture liquor of the *Euglena* isolate.

**Table 2:** **Changes in concentrations of DOC and monosaccharides in cell-free culture liquors from the *Chlorella* and *Euglena* isolates, inoculated with acidophilic heterotrophic bacteria**.

	***Acidiphilium* SJH**	**“*Ac. aromatica*” PFBC**	***Ab. capsulatum*^*T*^**	**Sterile control**
***CHLORELLA* CELL-FREE CULTURE LIQUOR**				
DOC (mg/L) Day 0 Day 6	167 155 ± 4	167 162 ± 2	167 158 ± 3	167 168 ± 1
Glucose (μM) Day 0 Day 6	5.0 < 1	5.0 5.0 ± 0.1	5.0 < 1	5.0 5.1 ± 0.4
Fructose (μM) Day 0 Day 6	38.5 < 1	38.5 < 1	38.5 < 1	38.5 38.7 ± 0.3
***EUGLENA* CELL-FREE CULTURE LIQUOR**				
DOC (mg/L) Day 0 Day 6	192 170 ± 1	192 1.0 ± 3.0	192 175 ± 4	192 193 ± 2
Glucose (μM) Day 0 Day 6	65 < 1	65 64.5 ± 1.3	65 < 1	65 64.1 ± 0.7
Mannitol (μM) Day 0 Day 6	43 < 1	43 42.4 ± 0.3	43 1.9 ± 0.2	43 43.7 ± 0.5

“±” represent range values of duplicate cultures.

## Discussion

Acidophilic micro-algae have widespread distribution in MIWs (Novis and Harding, 2007; Das et al., 2009), though they have been the focus of far less research than prokaryotic acidophiles. One reason for this is that eukaryotes are not directly involved in the geochemical transformations of iron and sulfur that result in the dissolution of sulfide minerals and which give rise to the formation of acid mine drainage waters, as is the case with chemolitho-autotrophic and chemolitho-heterotrophic acidophiles (Johnson and Hallberg, 2009). However, their indirect impact on these transformations can be considerable. Brake et al. (2001) reported that prolific growth of *E. mutabilis* can lead to MIWs being over-saturated (by up to 200%) with dissolved oxygen. Waters draining underground mines and adits are frequently devoid of dissolved oxygen, and soluble iron tends to be present predominantly as reduced ferrous iron (Johnson, 2003; Johnson et al., 2012). Oxygenation of MIWs by acidophilic algae facilitates the oxidation of ferrous iron and reduced sulfur compounds present in MIWs by bacteria such as *Acidithiobacilllus* and *Leptospirillum* spp., in reactions that are net proton-generating and which can result in further acidification of MIWs downstream of the point of discharge, as described for the Cantareras mine in Spain (Rowe et al., 2007). A second way in which algae can impact geochemical transformations in MIWs is by stimulating the growth of iron- and sulfur-reducing heterotrophic acidophiles via their organic exudates and lysates, as mine waters generally contain very small concentrations of DOC (Johnson, 2003; Das et al., 2009).

There have been many reports describing the global occurrence of *E. mutabilis* in MIWs, and this organism has frequently been considered as an “indicator species” of acid mine drainage (e.g., Valente and Gomez, 2007). This member of the Protista is a unicellular protozoan, and commonly referred to as an alga (Brake et al., 2001). In addition to being able to grow over a wide pH range (2.1–7.7; von Dach, 1943), *E. mutabilis* is also tolerant of elevated concentrations of total dissolved solids, including some transition metals and aluminium e.g., Novis and Harding, 2007; Valente and Gomez, 2007). The site from which the current isolate was obtained contains about 500 mg iron (ferrous plus ferric)/L and 50 mg copper/L (~0.8 mM), which are concentrations well below those which inhibited its growth *in vitro*. In contrast, there are few reports of *Chlorella* spp. in MIWs, and this alga has been reported to be more prevalent in acidic soils (Huss et al., 2002). Relatively little is known about the physiology of *C. protothecoides* var. *acidicola*, though Huss et al. (2002) reported that its lower pH and upper temperature limits for growth are 2.0 and 34°C, respectively, which are similar to those found in the present study.

Concentrations of DOC increased to similar extents during growth *in vitro* of both the *Euglena* and *Chlorella* isolates. Values obtained were similar to those found in sulfur-grown cultures of the chemolitho-autotrophic bacteria *Acidithiobacillus ferrooxidans* and *Acidithiobacillus caldus* (Ňancucheo and Johnson, 2010), though glycolic acid represented a greater proportion of DOC in *Chlorella* and *Euglena* cultures (12–14%) than in cultures of these two bacteria (both ~5%). The presumed origin of the glycolic acid in all these acidophiles is due to the activity of RuBisCO which, besides combining carbon dioxide and ribulose bisphosphate (RUBP) also oxidises RUBP to phosphoglyceric acid and phosphoglycolate. Enzymatic hydrolysis of the latter compound produces glycolate, which is exported out of actively growing cells (Fogg and Watt, 1965). In low pH liquors, glycolate is present as undissociated glycolic acid which, in common with acetic and many other small molecular weight aliphatic acids, is highly toxic to most acidophiles (Johnson and Hallberg, 2009). Production and excretion of glycolic acid has previously been reported for neutrophilic micro-algae (Miller et al., 1963; Fogg and Watt, 1965). Among acidophilic prokaryotes, the ability to metabolize glycolic acid appears to be restricted to *Firmicutes* (e.g., *Sulfobacillus* spp). None of the three species of heterotrophic acidophiles used in the present study were previously found to grow on this organic acid (Ňancucheo and Johnson, 2010).

Other organic compounds (monosaccharides) identified in cell-free culture liquors of the two algae have more widespread use by heterotrophic acidophiles (Johnson and Hallberg, 2009). *Acidiphilium* SJH and *Ab. capsulatum* metabolized fructose and glucose originating from *Chlorella*, and mannitol and glucose present in the *Euglena* culture, within 6 days. Mass balance calculations of DOC and sugar concentrations confirmed that other (unidentified) materials were also catabolized by the bacteria during this period. The situation with “*Ac. aromatica*” PFBC was very different. In contrast to other *Acidocella* spp, this candidate species grows on a restricted range of substrates, including fructose and some aliphatic acids, but not on glucose or mannitol (Gemmell and Knowles, 2000; Johnson and Hallberg, 2009). While it grew in cell-free culture liquors of *Chlorella*, only fructose (of the compounds analysed) was metabolized, and its failure to grow in cell-free liquor from *Euglena* was apparently due to neither mannitol nor glucose being suitable substrates for this acidophile.

One of the characteristics of the acidophilic algae studied was their abilities to take up (and presumably to metabolize) glycolic acid that they had previously excreted. This was more closely studied with the *Chlorella* isolate, where it was shown that extraneous glycolic acid added to a non-photosynthesizing culture was quantitatively removed within 3 days. The ability of some micro-algae to grow as heterotrophs has been widely documented, e.g., Miller et al. (1963) reported that a neutrophilic *Chlorella* (*C. pyrenoidosa*) also excreted glycolic acid into its growth medium when cultures were illuminated and that this was taken up rapidly by the algal cells in the absence of light.

The significance of acidophilic micro-algae in sustaining populations of acidophilic heterotrophic bacteria has been illustrated in studies that have compared macroscopic streamer/mat growth in acidic subterranean metal mines and those in sunlight-receiving acid drainage streams (Johnson, 2012). In subterranean sites, where primary production is mediated exclusively by chemolithotrophic acidophiles, streamer growths are often dominated by autotrophic prokaryotes (such as “*Ferrovum myxofaciens*”) whereas micro-algae growing on the surfaces of streamer/mat growths in sunlight-receiving promote the development of underlying microbial communities that are more dominated by heterotrophic acidophiles, including many (*Acidiphilium, Acidocella*, and *Acidobacterium* spp.) that catalyze the dissimilatory reduction of ferric iron. Elsewhere, Ňancucheo and Johnson (2011) found that sulfidogenesis was far more pronounced in mineral tailings mesocosms that were inoculated with acidophilic algae and acidophilic SRB (aSRB) than with aSRB alone. This provides further support for the hypothesis presented in the present study that acidophilic micro-algae stimulate the growth of acidophilic heterotrophic bacteria by providing them with organic substrates, and suggests that such interactions can be the basis of ecological engineering strategies for prevention and remediation of mine-related metal pollution.

### Conflict of interest statement

The authors declare that the research was conducted in the absence of any commercial or financial relationships that could be construed as a potential conflict of interest.
